# Laparoscopic resection of abdominal cystic lymphangioma derived from lesser omentum: Case report

**DOI:** 10.1097/MD.0000000000018641

**Published:** 2020-01-03

**Authors:** Yuhei Hamaguchi, So Arita, Naoko Sugimoto, Osamu Inamoto, Hidekazu Takagi, Masafumi Kogire, Toshiyuki Kitai

**Affiliations:** Department of Surgery, Kishiwada City Hospital, Kishiwada, Osaka 5968501, Japan.

**Keywords:** cystic lymphangioma, laparoscopy, preoperative diagnosis, surgical resection

## Abstract

**Rationale:**

Cystic lymphangiomas are uncommon congenital malformations that originate from lymphatic channels. Lymphangiomas frequently appear in the head, neck, and axillary regions of children. Abdominal cystic lymphangiomas are extremely rare, having a reported incidence of 1 in 20,000 to 250,000.

**Patient concerns:**

A 50-year-old female patient was admitted to our hospital with a cough that had persisted for several weeks. Abdominal ultrasonography incidentally revealed a multilocular cystic lesion in the lesser curvature of the stomach.

**Diagnosis:**

Preoperative findings indicated that the lesion was cystic lymphangioma. However, the possibility of a pancreatic tumor could not be completely excluded.

**Interventions:**

Laparoscopy revealed a multilocular cyst in the lesser curvature of the stomach. The gastrocolic ligament was divided, and the body and tail of the pancreas was exposed in the omental bursa, showing that the cystic lesion was not derived from the pancreas but from the lesser omentum. Although it was located directly beside the left gastric artery, the cyst was enucleated and totally resected laparoscopically without sacrificing the artery.

**Outcomes:**

The cystic lesion was histopathologically diagnosed as an abdominal cystic lymphangioma originating from the lesser omentum. The patient was discharged on the postoperative day 4 without complications.

**Lessons:**

Preoperative imaging cannot completely distinguish abdominal cystic lymphangiomas from other types of cystic tumors. Because cystic lymphangiomas have the potential to grow, invade vital structures, and develop life-threatening complications, laparoscopic assessment followed by total resection is considered a useful treatment strategy for peripancreatic cystic lesions.

## 1 Introduction

Cystic lymphangiomas are congenital benign tumors of lymphatic vessels that are disconnected from normal lymphatic systems.^[1]^ Lymphangiomas usually appear in the head, neck, and axillary regions, most often in children.^[2,3]^ Less than 1% of patients present with cystic lymphangiomas in the mesentery, greater omentum, and retroperitoneum.^[1,4–6]^ Among intraabdominal lymphangiomas, 85%, 20%, and 5% of them are derived from the small bowel mesentery, mesocolon, and retroperitoneum, respectively.^[7]^ Although most abdominal lymphangiomas are initially asymptomatic, symptoms including nausea, vomiting, and pain can gradually develop as they progress.^[3]^ Various modalities, such as abdominal ultrasonography (US), computed tomography (CT), and magnetic resonance imaging, can definitively diagnose cystic lymphangiomas.^[8,9]^ However, some peripancreatic cystic lymphangiomas are difficult to differentiate from pancreatic tumors, including mucinous cystic neoplasms or intraductal papillary mucinous neoplasms. This report describes a patient with an abdominal cystic lymphangioma derived from the lesser omentum that was difficult to preoperatively diagnose.

## 2 Case presentation

**Figure 1:**
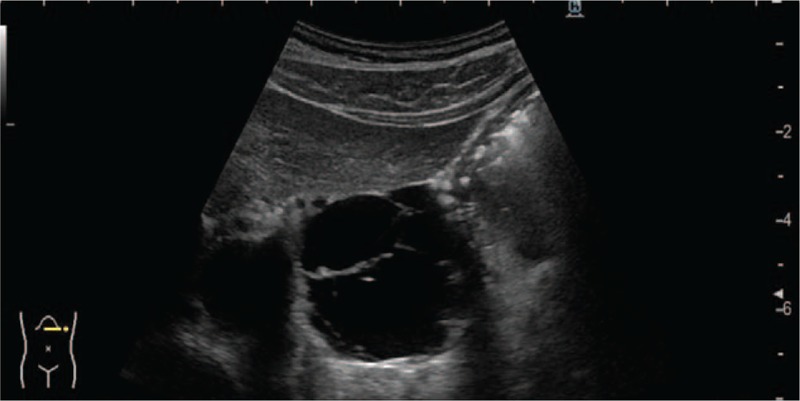
Abdominal ultrasonography findings. Multilocular cystic lesion is evident in lesser curvature of stomach.

**Figure 2:**
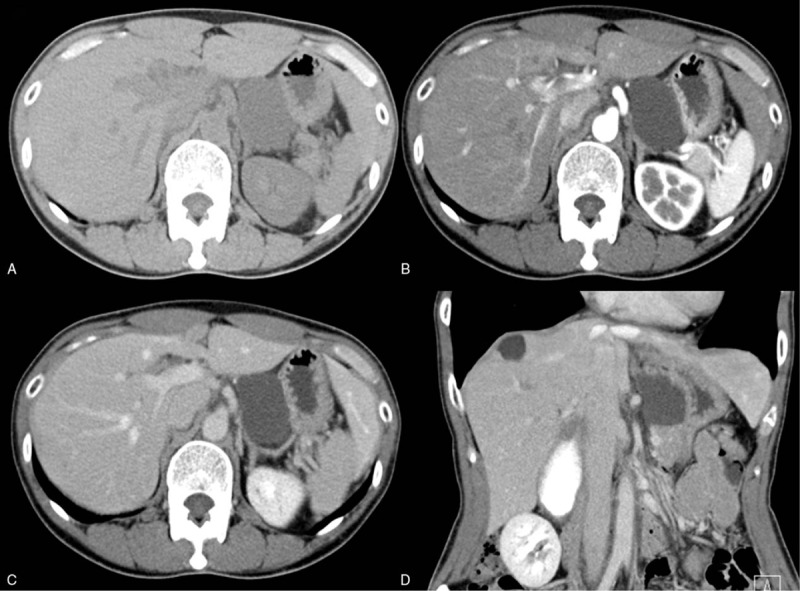
Multiphasic contrast-enhanced computed tomography findings. Unilocular cystic lesion in lesser curvature of stomach has no separation or mural nodules, and a thin wall without contrast enhancement. (A) Axial plain CT image, (B) Arterial phase and (C) delayed phase of the dynamic contrast study, and (D) Coronal CT image. CT = computed tomography.

**Figure 3:**
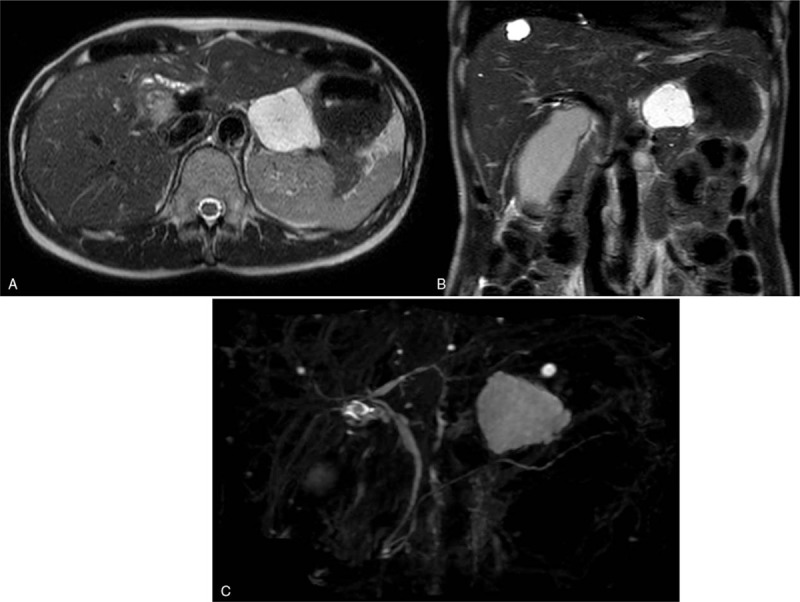
Magnetic resonance imaging findings. Multilobulated cyst with thin internal septum spreading out from pancreatic tail does not appear to communicate with main pancreatic duct. (A) Axial and (B) coronal image and (C) Magnetic resonance cholangiopancreatography.

**Figure 4:**
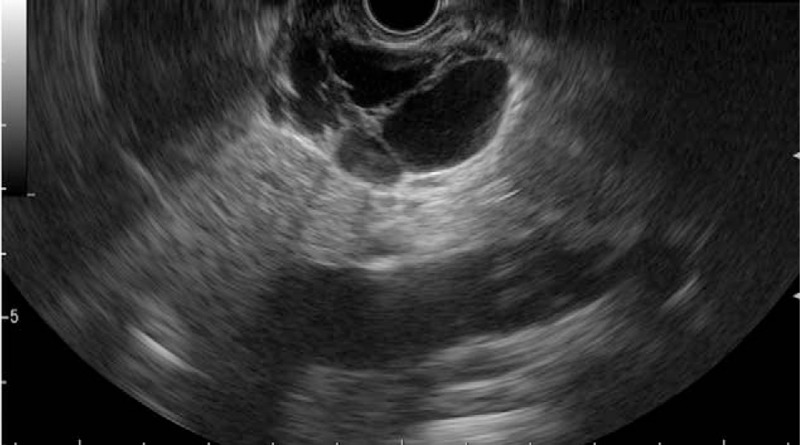
Endoscopic ultrasound findings. Multilocular cystic lesion along the pancreatic tail has thin multiple septum and mural nodules.

**Figure 5:**
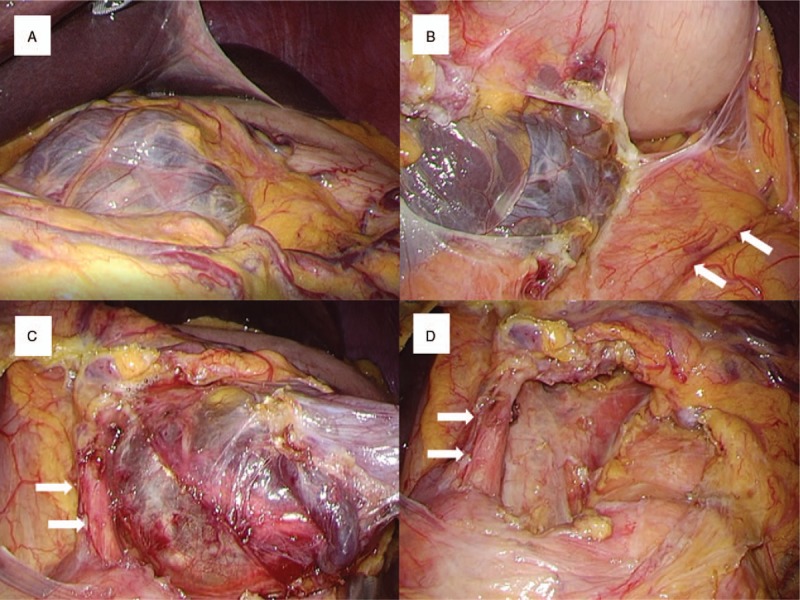
Laparoscopy findings. (A) Multilocular cyst is in lesser curvature of stomach. (B) Exposing pancreatic body and tail (arrow) reveal that cystic lesion is derived from lesser omentum. (C) The cyst is located directly beside the left gastric artery (arrow). (D) Enucleation proceeds without sacrificing the left gastric artery (arrow).

**Figure 6:**
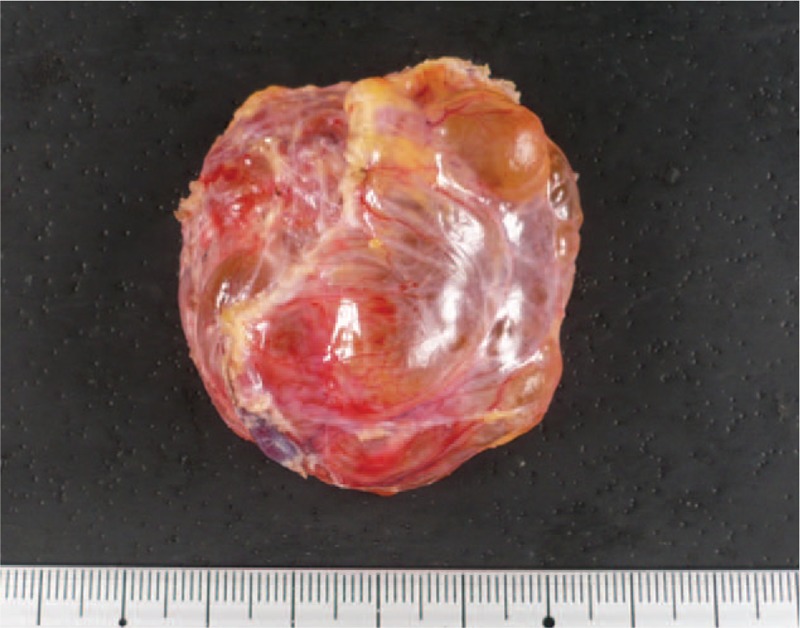
Macroscopic findings. Cyst measures 4.8 × 4.5 cm with multiple septae.

**Figure 7:**
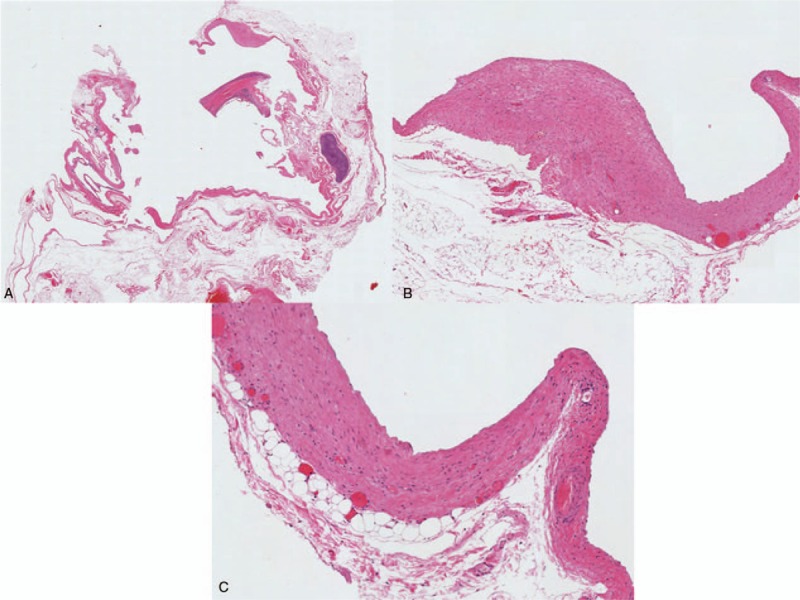
Microscopic findings. (A) Inner surface of cyst is lined with single layer of flattened cells without atypia and focal smooth muscle. (B) Hematoxylin and eosin (H&E) staining ×40, and (C) H&E staining ×100.

A 50-year-old female patient was admitted due to cough that had persisted for several weeks. Her past medical and surgical history was unremarkable. Laboratory data, chest X-ray, and chest CT did not reveal any significant findings. Abdominal US incidentally identified a 42 × 38 × 35-mm multilocular cystic lesion surrounded by the liver, stomach, left kidney, and pancreas (Fig. 1). Multiphasic contrast-enhanced CT imaging revealed that the cystic lesion was unilocular, without separation or mural nodules, and it was located in the lesser curvature of the stomach (Fig. 2). A thin cyst wall had no contrast enhancement. A multilobulated cyst with a thin internal septum spread outward from the pancreatic tail on MR images, but did not appear to communicate with the main pancreatic duct (Fig. 3). Endoscopic US showed a multilocular cystic lesion along the pancreatic tail with a thin multiple septum and mural nodules (Fig. 4). These findings could not confirm that the cystic lesion was derived from the pancreas, and a mucinous cystic neoplasm was a possibility considering her age and sex. Therefore, she provided written, informed consent to undergo laparoscopic resection. Laparoscopy confirmed the multilocular cyst in the lesser curvature of the stomach (Fig. 5). The gastrocolic ligament was divided, and the body and tail of the pancreas was exposed in the omental bursa. Further observation revealed that the cystic lesion was derived from the lesser omentum. Although the cyst was located directly beside the left gastric artery, enucleation proceeded without sacrificing the artery. Macroscopically, the 4.8 × 4.5-cm cyst had multiple septae (Fig. 6). Microscopically, the inner surface of the cyst was lined by a single layer of flattened cells without atypia and focal smooth muscle (Fig. 7). The lesion was diagnosed as cystic lymphangioma. The patient was discharged on postoperative day 4 without complications. The patient was free of recurrence after 1 year of follow-up.

Informed written consent was obtained from the patient for publication of this case report and accompanying images.

## 3 Discussion

Lymphangiomas are rare congenital malformations that originate from lymphatic channels.^[1]^ Abdominal lymphangiomas are extremely rare with a reported incidence of 1 in 20,000 to 250,000.^[10,11]^ Although lymphangiomas can occur at any age, there is a bimodal distribution in age at presentation; >80% of patients are below the age of 5 years, and the remainder are aged around 40 years.^[10,12,13]^ Although the exact etiology of lymphangiomas remains obscure, they might be associated with congenital anomalies of the lymphatics during embryological development, or they might arise as the result of lymphatic obstruction secondary to bleeding or inflammation of the lymphatic channels.^[1,14,15]^ The latter mechanism is more likely related to adult manifestations.^[16]^ Lymphangiomas pathologically comprise simple capillary, cavernous, and cystic types.^[1,17,18]^ Of these, the cystic type is found in the abdominal cavity, and typically occurs in spaces surrounded by loose connective tissue such as the mesentery and retroperitoneum.^[1]^

Abdominal US and CT are helpful for detecting cystic lesions and for defining the extent of masses. US is a sensitive and specific modality to evaluate the location and size of cysts.^[1,9]^ The typical appearance of lymphangiomas on US is a well-circumscribed lesion with multiple thin septa and solid echogenicity with a honeycomb pattern.^[9]^ Abdominal CT is more sensitive and useful to differentiate lymphangiomas from other abdominal cysts.^[13,19]^ CT imaging of a cystic lymphangioma generally demonstrates a unilocular or multilocular mass with enhanced thin septae and wall.^[9,20]^ CT and magnetic resonance imaging can add more information regarding the location, size, other organ involvement, and solid components within cysts, which can contribute to preoperative surgical planning.^[1]^ Although others have described the imaging features of lymphangioma and other abdominal cystic tumors,^[13,21]^ preoperative diagnoses based on imaging findings alone are usually not definitive. The cystic lesion in our patient was most likely to be a cystic lymphangioma according to preoperative imaging. However, the border between the cystic lesion and the pancreas was unclear, and we could not completely determine whether the cyst was derived from the pancreas. Therefore, considering the possibility of pancreatectomy, we initially proceeded with laparoscopy, which revealed that the cyst originated from the lesser omentum.

Complete resection with negative margins on microscopy is the standard treatment for cystic lymphangiomas.^[1]^ Although these tumors are generally benign, they tend to invade adjacent organs and recur after curative resection,^[12,22]^ and large infiltrative tumors that involve vital structures are difficult to completely resect. Recurrence rates after incomplete and even complete resection are 35% to 64% and 17% to 24%, respectively.^[1,23,24]^ On the other hand, postoperative complications including infection, bleeding, and neurovascular injury occur in 33% of patients after surgical resection.^[12]^ Simple aspiration followed by injection of sclerosing agents is considered ineffective because of infection and recurrence rates are high.^[20]^ However, sclerotherapy has become the mainstay of treatment for macrocystic lesions because it is more effective than surgical resection and morbidity rates are lower.^[25,26]^ Madsen et al^[26]^ described 10 patients in whom abdominal lymphangiomas were completely resolved by doxycycline sclerotherapy, and 83.3% of them did not have recurrence. Although sclerotherapy might be a better treatment strategy, our patient underwent laparoscopic resection for a total biopsy, because preoperative assessment using various modalities could not completely exclude the possibility of a pancreatic tumor.

In conclusion, we described a patient with a rare, abdominal cystic lymphangioma derived from the lesser omentum. Preoperative imaging modalities have limited capacity to distinguish cystic lymphangiomas from pancreatic tumors. Abdominal cystic lymphangiomas have the potential to grow, invade vital structures, and develop life-threatening complications. Therefore, laparoscopic exploration followed by total resection seems a reasonable strategy for treating peripancreatic cystic lesions that is less invasive than laparotomy.

## Author contributions

**Conceptualization:** Yuhei Hamaguchi.

**Data curation:** Yuhei Hamaguchi, So Arita, Naoko Sugimoto, Osamu Inamoto, Hidekazu Takagi.

**Investigation:** Yuhei Hamaguchi, Toshiyuki Kitai.

**Supervision:** Masafumi Kogire, Toshiyuki Kitai.

**Writing – original draft:** Yuhei Hamaguchi, Toshiyuki Kitai.

**Writing – review & editing:** So Arita, Naoko Sugimoto, Osamu Inamoto, Hidekazu Takagi, Masafumi Kogire.
